# Telemonitored standardized titration for heart failure with reduced ejection fraction, an open clinical cohort study

**DOI:** 10.1093/ehjdh/ztaf062

**Published:** 2025-06-05

**Authors:** Antros Louca, Daniel Thomas, Karin Odefjord, Rami Genead, Charlotte Nordberg Backelin, Charlotta Ljungman, Kristofer Skoglund, Entela Bollano, Araz Rawshani, Helén Sjöland, Niklas Bergh, Tomas Mellberg

**Affiliations:** Department of Molecular and Clinical Medicine, Institute of Medicine, Sahlgrenska Academy, University of Gothenburg, Gothenburg, Sweden; Department of Cardiology, Sahlgrenska University Hospital, Blå Stråket 3, Gothenburg 413 46, Sweden; Department of Molecular and Clinical Medicine, Institute of Medicine, Sahlgrenska Academy, University of Gothenburg, Gothenburg, Sweden; Department of Cardiology, Sahlgrenska University Hospital, Blå Stråket 3, Gothenburg 413 46, Sweden; Department of Medicine, South Älvsborg Hospital, Borås, Sweden; Department of Molecular and Clinical Medicine, Institute of Medicine, Sahlgrenska Academy, University of Gothenburg, Gothenburg, Sweden; Department of Cardiology, Sahlgrenska University Hospital, Blå Stråket 3, Gothenburg 413 46, Sweden; Department of Molecular and Clinical Medicine, Institute of Medicine, Sahlgrenska Academy, University of Gothenburg, Gothenburg, Sweden; Department of Cardiology, Sahlgrenska University Hospital, Blå Stråket 3, Gothenburg 413 46, Sweden; Department of Molecular and Clinical Medicine, Institute of Medicine, Sahlgrenska Academy, University of Gothenburg, Gothenburg, Sweden; Department of Cardiology, Sahlgrenska University Hospital, Blå Stråket 3, Gothenburg 413 46, Sweden; Department of Molecular and Clinical Medicine, Institute of Medicine, Sahlgrenska Academy, University of Gothenburg, Gothenburg, Sweden; Department of Cardiology, Sahlgrenska University Hospital, Blå Stråket 3, Gothenburg 413 46, Sweden; Department of Molecular and Clinical Medicine, Institute of Medicine, Sahlgrenska Academy, University of Gothenburg, Gothenburg, Sweden; Department of Cardiology, Sahlgrenska University Hospital, Blå Stråket 3, Gothenburg 413 46, Sweden; Department of Molecular and Clinical Medicine, Institute of Medicine, Sahlgrenska Academy, University of Gothenburg, Gothenburg, Sweden; Department of Medicine, Geriatrics and Emergency Medicine, Sahlgrenska University Hospital/Östra, Gothenburg, Sweden; Department of Molecular and Clinical Medicine, Institute of Medicine, Sahlgrenska Academy, University of Gothenburg, Gothenburg, Sweden; Department of Diabetes and Cardiology, Angered Hospital, Gothenburg, Sweden; Department of Molecular and Clinical Medicine, Institute of Medicine, Sahlgrenska Academy, University of Gothenburg, Gothenburg, Sweden; Department of Cardiology, Sahlgrenska University Hospital, Blå Stråket 3, Gothenburg 413 46, Sweden

**Keywords:** Heart failure, Telemonitoring, GDMT titration

## Abstract

**Aims:**

To evaluate feasibility, efficacy, and safety of standardized medical titration at home using telemonitoring. Treatment for heart failure with reduced ejection fraction (HFrEF) has advanced rapidly, emphasizing swift initiation and titration of guideline-directed medical therapy (GDMT) to improve outcomes. Implementing this in practice remains a significant challenge for healthcare. This study proposes a standardized home-based titration process incorporating home-based monitoring (HBM) to enhance GDMT titration, reduce delays, and limit the need for in-clinic assessment visits.

**Methods and results:**

60 patients were enrolled in this open cohort study. Standardized pre-specified titration schedules in combination with HBM were evaluated. Outcome measures included the time to optimal medical therapy (OMT), doses of GDMT at 8 weeks and 6 months, and safety evaluation through adverse events. The median time to OMT was 48 days (IQR 42–60). All participants achieved OMT within 6 months. At 8 weeks, 73%, 85%, and 88% had reached target doses for beta-blockers, ACE inhibitors, and mineral receptor antagonists, respectively. All participants reached SGLT2i target dosage. By 6 months, 62%, 73%, 80%, and 97% were on target doses for these medications, and 43% had achieved target doses for all four GDMT drugs. No serious adverse events occurred during titration.

**Conclusion:**

We present a novel and promising approach for achieving OMT and high GDMT doses in patients with HFrEF. The utilization of standardized protocols has the potential to optimize the titration process of GDMT, and with HBM support, it can be accomplished with few in-clinic visits.

## Graphical Abstract

**Graphical Abstract:**
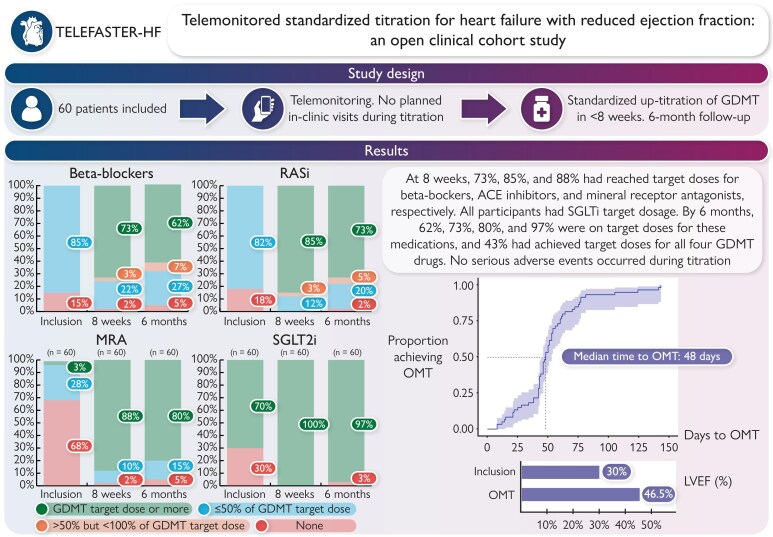

## Introduction

Heart failure (HF) is a condition with high morbidity and mortality, often leading to worse outcomes than many cancers.^1^ It remains a leading cause of hospital admissions in the USA and Europe, with high readmission rates strongly predicting mortality.^2-5^ Current guidelines from Europe and the USA recommend a four-pillar approach for treating heart failure with reduced ejection fraction (HFrEF), including three guideline-directed medical therapy (GDMT) classes with sequential titration.^6,7^ However, despite its proven ability to reduce all-cause mortality by up to 61% compared with placebo, numerous studies indicate that HF patients are often sub-optimal treated.^8-10^ Slow and ineffective up-titration, with few patients reaching target doses, is associated with increased morbidity, higher rates of rehospitalization, and a poorer prognosis.^4,8,9,11^ These gaps in care highlight the need for innovative and effective strategies to optimize HF management and improve patient outcomes.

The STRONG-HF study compellingly demonstrated the importance of rapid GDMT up-titration, reducing symptoms, HF readmissions, and mortality in acute HF patients.^12^ At the same time, home-based monitoring (HBM) is gaining attention as a strategy to optimize heart failure treatment. Emerging evidence suggests that HBM can further reduce all-cause mortality and hospitalizations compared with usual care.^13-15^ Combining HBM and titration of GDMT may offer an alternative approach to optimizing HF treatment.^16,17^ Other encouraging strategies for GDMT improvement using HBM have involved digital consultations and clinical support using guideline-based digital summaries.^18^ However, studies integrating these methods have often involved multiple individual clinical decisions throughout the titration process, which is likely to impact both the time to reach optimal medical therapy (OMT) and the proportion of patients achieving GDMT. A potentially more effective strategy could be to pair HBM with a standardized medical titration protocol, thereby bypassing the need for continuous clinical decision-making and allowing titration to proceed uninterrupted *unless* a medical adjustment is genuinely necessary.

The TELEmonitored FAst Standardized Titration for Efficient Response in Heart Failure with reduced ejection fraction (TELEFASTER-HF) study evaluated the safety and feasibility of a standardized 8-week up-titration plan with no planned clinical visits or phone consultations, supported by HBM, in newly diagnosed HF patients.

## Methods

### Study design and patients

The TELEFASTER-HF study was a multi-centre, open clinical cohort trial designed to evaluate the feasibility, efficacy, and safety of a standardized medication up-titration protocol, aiming for target doses of GDMT within 8 weeks. This protocol was supported by HBM and focused on patients with newly diagnosed HF. The safety of up-titration was assessed by HBM and pre-determined regulatory laboratory assessments, without any planned in-clinic visits.

Participants were recruited between December 2022 and February 2024 at three regional cardiology and internal medicine clinics—Sahlgrenska University Hospital (Sahlgrenska), Sahlgrenska University Hospital (Östra), and Södra Älvsborg Hospital (Borås) in the region of Västra Götaland in Sweden. Patients diagnosed with HFrEF, within the preceding 3 months were invited to participate. Newly diagnosed patients included those hospitalized for decompensated HF and stable patients referred to the outpatient HF clinic. Patient identification was conducted in HF outpatient clinics and inpatient hospital wards. A dedicated HF nurse at each site then performed an eligibility screening consultation, after which the patient was finally offered inclusion in the study during a visit with a cardiologist.

Exclusion criteria included estimated glomerular filtration rate (eGFR) < 45 mL/min, current treatment with beta-blockers (BB) or renin–angiotensin system inhibitors (RASi) at >50% of target dose, contraindications to any of the four key HF drug classes, lack of smartphone or internet access, and inability to handle remote monitoring or give informed consent. All participants met with a cardiologist and an HF nurse for a clinical evaluation at inclusion. Medical history, medications, and diagnosis were reviewed, and participants were informed about their heart failure diagnosis, treatment strategy, and any planned investigations. Alcohol consumption was assessed based on weekly standard units’ intake, following Swedish National Board of Health and Welfare guidelines for risky consumption. According to these guidelines, alcohol consumption exceeding 10 standard units per week is considered overconsumption or risky use.

This study is registered at ClinicalTrials.gov with trial registration number NCT05637853.

### Home-based monitoring

**Figure 1:**
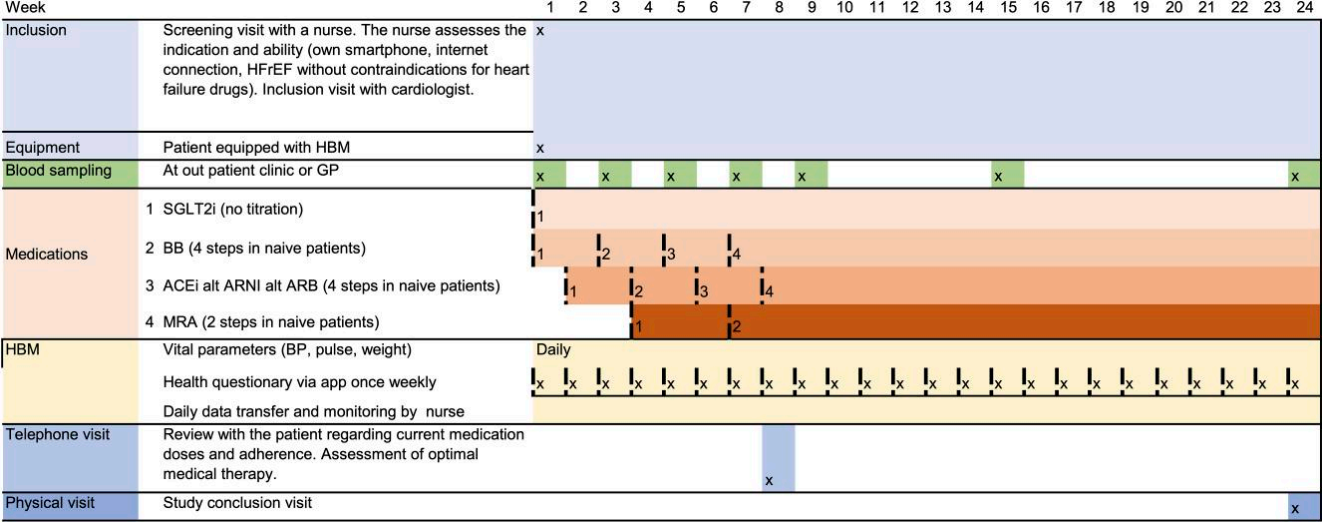
Study overview. Summary of the workflow for home-based monitoring, standardized medical titration, and blood sampling in the TELEFASTER-HF study. Guideline-directed medical therapy (GDMT) titration followed standardized protocols, adjusted to individual regimens, advancing to target doses, or modified for side effects. GP, general practitioner.

Participants were provided with a Bluetooth-enabled blood pressure monitor (A&D medical UA-656 BLE, Japan, CE 0123) and a digital scale (Wunder W1090, Italy, CE MDR) linked to a smartphone application (Platform 24), automatically transmitting daily measurements of weight, blood pressure, and pulse to the HF clinic. Additionally, participants completed a weekly health questionnaire to assess their symptoms, including dyspnoea, leg oedema, and fatigue (see Supplementary material online, table  *S2*). Blood samples were obtained at baseline and subsequently every 2 weeks during the first 9 weeks, with additional collections at weeks 15 and 24. The samples were collected at the most convenient facility for each participant. Participants had the freedom to choose their preferred location, whether at a general practitioner’s office or a hospital. The HF nurses issued digital referrals for blood sampling, which were accessible to the designated laboratory unit. The tests performed in the study included plasma high sensitive Troponin I (P-hsTnI), plasma N-terminal pro-brain natriuretic hormone (P-NT-proBNP), P-Sodium, P-Potassium, P-Creatinine, Pt-eGFR (creatinine) relative, blood haemoglobin (B-Hb), blood white blood cell count (B-WBC), B-Platelets, plasma high sensitive C-reactive protein (P-hsCRP), plasma alanine aminotransferase (P-ALT), plasma aspartate aminotransferase (P-AST), and P-Glucose. A telephone consultation with a cardiologist was scheduled at 8 weeks to evaluate medication adherence, adverse events, and the progression towards OMT (*Figure 1*). At 6 months, participants attended a final in-person evaluation at the clinic.

Throughout the study, participants had access to a chat function within the app, enabling direct communication with a devoted HF nurse. This feature allowed timely responses to participant inquiries, reminders for medical titration, and the flexibility for clinical evaluations by a HF nurse and/or cardiologist when necessary.

No study-specific visits or consultations required payment. After the final visit at 6 months, participants continued follow-up according to the standard of care.

### Standardized medical titration

The study protocol included a standardized sequential titration of GDMT with pre-specified titration schemes tailored to align with each participant’s baseline medical regimen (see supplementary material online, tables  *S3*–*S11*). Titration proceeded at the designated pace until full target doses were achieved or side effects necessitated alterations. The study adhered to the 2021 ESC guidelines for HFrEF regarding specified GDMT and target dose definitions, and any adjustments to the titration plan were made according to clinical practice.^6^ Participants not already on sodium–glucose cotransporter 2 inhibitors (SGLT2i) treatment were started on either dapagliflozin or empagliflozin at target doses immediately after study inclusion combined with either the initiation or an increase in beta-blocker treatment. In the second week, RASi—angiotensin converting enzyme inhibitors (ACEi), angiotensin receptor-neprilysin inhibitor, or angiotensin II receptor blockers (ARB)—were initiated or up-titrated as indicated by each participant’s prior treatment. In subsequent weeks, BB and RASi were alternately up-titrated according to the standardized protocol. MRA were introduced and/or increased in weeks 3 and 7, depending on prior ongoing dosage. Participants already on BB, RASi, or MRA at inclusion continued their initial doses until the titration algorithm indicated an increase (*Figure 1*).

OMT was defined as the highest individually tolerable doses of GDMT. OMT was clinically evaluated by a cardiologist, and the doses had to be tolerated for at least 2 weeks following dose escalation.

### Outcome measures

The primary outcomes were time to OMT and the doses achieved of the four HF drug classes at 8 weeks and 6 months, respectively.

Secondary outcomes included time to first event for all-cause hospitalization, all-cause mortality, and hospitalization due to HF exacerbation. Furthermore, changes in key clinical variables and biomarkers were assessed—such as weight, systolic blood pressure (SBP), NT-proBNP, and left ventricular ejection fraction (LVEF)—between inclusion and 6 months. Echocardiography was performed by expert echocardiographers at each hospital’s clinical physiology department. LVEF was calculated via the Biplane Simpson method.

Safety was evaluated through the incidence of adverse events.

### Consent

The study was conducted in compliance with the ICH Good Clinical Practice Guidelines and per the ethical principles of the Declaration of Helsinki and was approved by the Swedish Ethical Review Authority (registration number 2020-02632 with amendment 2022-01899-02). Oral and written consent was collected from all participants.

### Statistical analysis

Continuous variables are presented as median values with inter-quartile range unless otherwise specified. Categorical variables are presented as both counts and percentages. Dosage of GDMT was treated as an ordinal variable with four categories: None, ≤50% of the target dose, >50% but <100% of the target dose, and ≥100% of the target dose (see Supplementary material online, *Table S1*). To analyse the effects of the intervention on dose titration, an ordinal generalized estimating equations model was utilized, which was selected to account for the clustered nature of repeated measurements within subjects over time, providing robust standard errors. A cumulative logit link was used to model the probability of transitions between the four dose categories across three time points (inclusion, 8 weeks, and 6 months).

For binary dose outcomes, such as SGLT2i, where patients either achieved target dose or no dose, we used penalized logistic regression with Firth’s correction. To evaluate the time to OMT, we used cumulative incidence analysis to plot the cumulative proportion of patients reaching OMT over the study period.

Changes in key clinical variables, such as weight, SBP, NT-proBNP levels, and LVEF, from inclusion to 6 months were analysed using the Wilcoxon signed-rank test (CI for standardized effect size).

Two-sided *P*-values of <0.05 were considered statistically significant. All analyses were performed using R (R Foundation for Statistical Computing, version 4.3.2).

## Results

### Baseline characteristics

Sixty patients participated in the study, with 28 (47%) hospitalized for acute heart failure and 32 (53%) enrolled as outpatients with HF diagnosed within 3 months. The median age at diagnosis was 62 years (IQR: 53–72), with a predominance of males (70%). The time from diagnosis of HF to inclusion in the trial was 21.5 days (IQR 6.5–33.25 days). The baseline characteristics of participants at inclusion are summarized in *Table 1*. The most common causes of HF were ischaemic heart disease (22%), dilated cardiomyopathy (22%), and arrhythmia (19%), with atrial fibrillation accounting for most arrhythmia cases (10 out of 11). One patient had an ICD as secondary prevention following myocardial infarction with subsequent cardiac arrest. No participants had a pacemaker. Forty-seven percent (*n* = 28) of participants had a diagnosis of hypertension, and 13% (*n* = 8) of participants had a diagnosis of diabetes mellitus type II before joining the study. Most participants initiated GDMT medication around the time of diagnosis. At inclusion, 85% of participants were on BB treatment (≤50% of the target dose) while 70% were on the target dose of SGLT2i. Eighty-two percent were receiving RASi at ≤ 50% of the target dose. Sixty-eight percent of participants were not receiving any MRA, and 3.3% were on target dose.

**Table 1:** Baseline characteristics of the TELEFASTER-HF cohort

Characteristic	TELE FASTER HF *N* = 60^a^	Missing
Age at diagnosis	62 (53, 72)	0
Sex		0
Male	42 (70%)	
Female	18 (30%)	
BMI (kg/m^2^)	26.0 (24.3, 30.3)	0
Smoking		0
Former smoker	30 (50%)	
Never	22 (37%)	
Current smoker	8 (13%)	
Alcohol habits		1
Normal consumption	37 (63%)	
Current overconsumption	12 (20%)	
Earlier overconsumption	6 (10%)	
Never drinks	4 (6.8%)	
Heart failure status		0
Acute heart failure	28 (47%)	
Outpatient	32 (53%)	
Heart failure aetiology		0
Ischaemic heart disease	13 (22%)	
Dilated cardiomyopathy	13 (22%)	
Atrial fibrillation/flutter	10 (17%)	
Other arrhythmia	1 (2%)	
Hypertensive cardiomyopathy	8 (13%)	
Other	3 (5%)	
Chemotherapy mediated	3 (5%)	
Valvular heart disease	3 (5%)	
Alcoholic cardiomyopathy	2 (3%)	
Undefined	4 (7%)	
Comorbidities		
Hypertension	28 (47%)	0
Diabetes mellitus type II	8 (13%)	0
Chronic pulmonary disease	8 (13%)	0
Atrial fibrillation/atrial flutter	23 (38%)	0
Medical history		
Previous cardiac arrest	2 (3%)	0
Previous myocardial infarct	9 (15%)	0
History of valvular heart disease	4 (7%)	0
History of revascularization	11 (18%)	0
LVEF (%)	30 (24, 35)	0
NYHA functional class		0
1	5 (8%)	
2	33 (55%)	
3	22 (37%)	
4	0 (0%)	
Electrocardiographic characteristics on inclusion		
ECG		0
Sinus rhythm	45 (75%)	
Atrial fibrillation/flutter	14 (23%)	
Other	1 (2%)	
LBBB	11 (18%)	0
QRS width	102 (92, 126)	0
Laboratory values on inclusion		
NTproBNP	1355 (670, 3220)	0
Creatinine	84 (73, 95)	1
eGFR	69 (61, 83)	1
Potassium	4.10 (4.00, 4.30)	1
Natrium	139.0(138.0, 141.0)	1
Oral medication on inclusion		
Beta-blocker		0
None	9 (15%)	
≤50% of target dose	51 (85%)	
SGLT2i		0
None	18 (30%)	
Target dose or more	42 (70%)	
RASi		0
None	11 (18%)	
≤50% of target dose	49 (82%)	
MRA		0
None	41 (68%)	
≤50% of target dose	17 (28%)	
Target dose or more	2 (3%)	
Statin	27 (45%)	0
Antiplatelet	15 (25%)	0
Anticoagulation	23 (38%)	0

^a^Median (IQR), frequency (%).

### Primary outcomes

**Figure 2:**
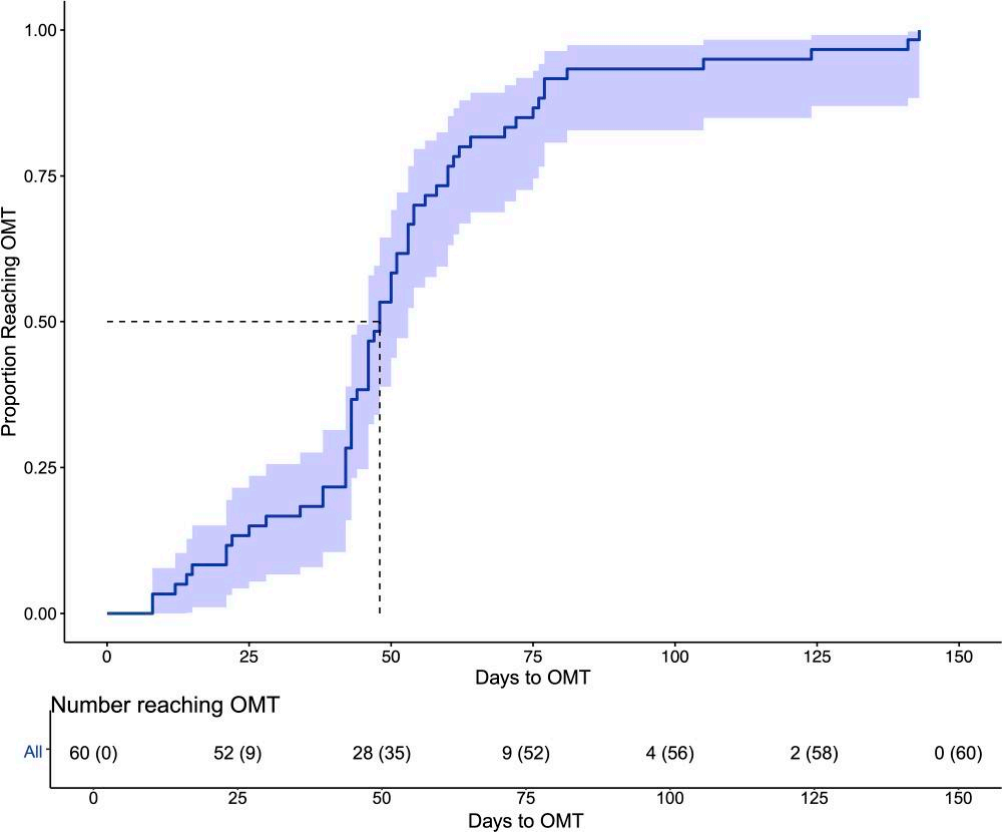
Time to optimal medical therapy (OMT). Cumulative proportion of patients achieving OMT over time. The median time to reach OMT from inclusion was 48 days (IQR 42–60 days). IQR, inter-quartile range.

All 60 participants successfully achieved OMT within 6 months. The median time to OMT from the day of inclusion was 48 days (IQR 42–60 days) (*Figure 2*).

**Figure 3:**
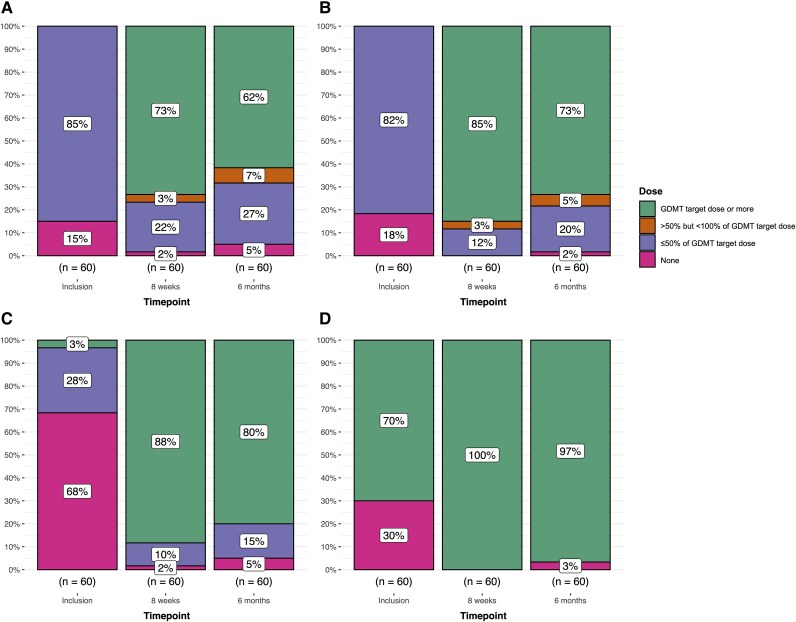
Titration of guideline-directed medical therapy (GDMT). Doses of beta-blockers (*A*), RASi (*B*), MRA (*C*), and SGLT2i (*D*) at inclusion, at 8 weeks, and 6 months. By 8 weeks, 73% of participants reached beta-blocker (BB) target doses, decreasing to 62% at 6 months, with 5% off BB therapy. For RASi, 85% achieved target doses by 8 weeks, decreasing to 73% at 6 months, with 2% off therapy. MRA target doses were reached by 88% at 8 weeks and 80% at 6 months, while 5% were off therapy. SGLT2i therapy increased from 70% at inclusion to 100% at 8 weeks, with 97% remaining on therapy at 6 months.

Within 8 weeks, 73% of participants had reached BB target doses. Only 2% were intolerant to BB therapy (*Figure 3*). By 6 months, participants on target BB doses declined to 62%, and 5% were without BB therapy. The OR for receiving an increased dose of BB between inclusion to 8 weeks was 57.8 (95% CI: 14.2–236.1), *P* < 0.001. Between 8 weeks and 6 months, BB dosage decreased significantly with some participants requiring dose reductions (OR: 0.6, 95% CI: 0.4–0.9, *P* = 0.01) after clinical reassessment (*Table 2*).

**Table 2:** Dose adjustments of HF medications over time

Medication	Time point comparison	Odds ratio [95% CI]	*P*-value
Beta-blockers	Inclusion|8 weeks	57.8 [14.2, 236.1]	<0.001
	Inclusion|6 months	34.5 [8.4, 141.6]	<0.001
	8 weeks|6 months	0.6 [0.4, 0.9]	0.01
Renin–angiotensin system inhibitors	Inclusion|8 weeks	506.3 [67.5, 3799.3]	<0.001
	Inclusion|6 months	235.1 [26.0, 2123.7]	<0.001
	8 weeks|6 months	0.5 [0.23, 0.97]	0.04
Mineralocorticoid receptor antagonist	Inclusion|8 weeks	151.9 [47.4, 486.9]	<0.001
	Inclusion|6 months	71.3 [23.2, 218.7]	<0.001
	8 weeks|6 months	0.5 [0.2, 0.9]	0.03
Sodium–glucose cotransporter inhibitor	Inclusion|8 weeks	52.7 [6.9, 677.0]	<0.001
	Inclusion|6 months	10.2 [3.0, 52.9]	<0.001
	8 weeks|6 months	0.2 [0.001, 2.4]	0.2

Odds ratios for dose adjustments of GDMT at inclusion, 8 weeks, and 6 months.

Eighty-five percent of participants achieved target RASi doses by 8 weeks, and no participants remained without RASi therapy. At 6 months, 73% were on target doses, and 2% were without RASi therapy. There was a significant increase in RASi dosage from inclusion to 8 weeks (OR: 506.3, 95% CI: 67.5–3799.3, *P* < 0.001) and from inclusion to 6 months (OR: 235.1, 95% CI: 26.0–2123.7, *P* < 0.001). From 8 weeks to 6 months, there was a decrease in the odds of maintaining higher dose categories (OR: 0.5, 95% CI: 0.23–0.97, *P* = 0.04), *Figure 2* and *Table 2*.

By 8 weeks, 88% of participants were on target doses of MRA and 10% on 50% of target doses. Only 2% remained without MRA therapy. By 6 months, 80% remained on target doses, 15% on half doses, and 5% were not receiving MRA therapy. There was a decrease in the odds of remaining on a higher dose category from 8 weeks to 6 months (OR: 0.5, 95% CI: 0.25–0.90, *P* = 0.02), *Figure 2* and *Table 2*.

Seventy percent of participants were on SGLT2i at inclusion. By 8 weeks, all participants (100%) were on SGLT2i therapy at target doses, and at 6 months, 97% remained on therapy. There was a substantial increase in SGLT2i use by 8 weeks (OR: 52.7, 95% CI: 6.9–677.0, *P* < 0.001), with no significant further change between 8 weeks and 6 months (OR: 0.2, 95% CI: 0.001–2.4, *P* = 0.2) (*Figure 2* and *Table 2*). Twenty-eight percent (*n* = 17) of participants changed their GDMT doses between 8 weeks and 6 months. Out of these, 76% (13 out of 17) had their GDMT doses altered during the follow-up period after achieving OMT. Dose reductions after initial OMT were primarily related to intolerable bradycardia in 54% (7 out of 13) and hypotensive side effects in 31% (4 out of 13).

At 6 months, 26 participants (43%) were receiving all four GDMT medications at target doses.

### Secondary outcomes

**Figure 4:**
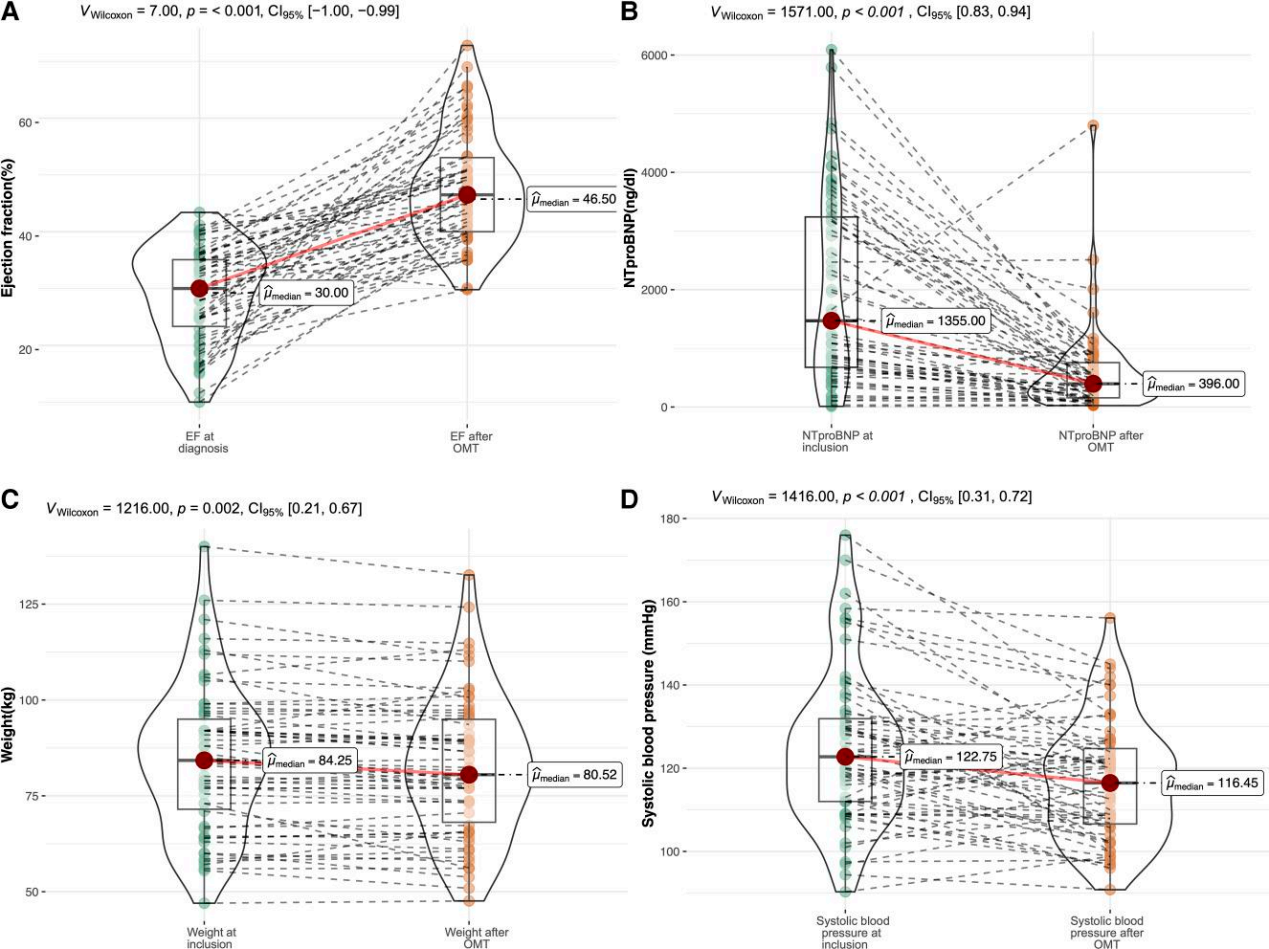
Changes in ejection fraction, NT-proBNP, weight, and blood pressure following optimal medical therapy (OMT). Comparison of ejection fraction (EF) at diagnosis and after achieved OMT (*A*), and NT-proBNP levels (*B*), weight (*C*), and systolic blood pressure (*D*) measured at inclusion and at 6 months, by which time all patients had achieved OMT. After OMT, follow-up echocardiography showed improved LVEF (median 46.5% vs. 30%, *P* < 0.001) compared with LVEF at diagnosis. NT-proBNP levels significantly decreased from 1355 to 396 pg/mL (*P* < 0.001), along with reductions in body weight (from 84.8 to 80.5 kg, *P* < 0.001) and systolic blood pressure (from 123 to 116 mmHg, *P* < 0.001).

Follow-up echocardiography performed after OMT showed significant improvement in LVEF, with a median ejection fraction of 46.5%, compared with 30% at the time of diagnosis (*P* < 0.001). The median NT-proBNP levels were significantly lower after OMT than at inclusion (396 pg/mL vs. 1.355 pg/mL, *P* < 0.001). The participants demonstrated a reduction in median body weight after OMT, from 84.3 to 80.5 kg (*P* < 0.001). Median SBP decreased from 122.8 to 116.5 mmHg (*P* < 0.001) (*Figure 4*). No deaths or hospitalizations for heart failure within 180 days from inclusion were reported.

### Health care resources

A clinical visit with a cardiologist was scheduled to coincide with the inclusion visit and following the study completion visit. HF nurses had dedicated time for telemonitoring surveillance and for managing urgent concerns through the chat function with participants (30 min daily in total for all participants). There was one designated cardiologist at each site responsible for questions from HF nurses regarding study-related issues (mainly titration-related questions). Daily data transfer was handled by one designated nurse at each site. Weekly rounds lasting 30 min to one hour, mainly involving titration issues and OMT evaluations, were conducted with a cardiologist. HF nurses also had an additional 3 h per week devoted to telemonitoring administration, blood sampling registration, and addressing non-urgent participant questions through the chat function. In-clinic assessments and telephone consultations were arranged as needed throughout the study. Over 6 months, 78% of participants had no clinical assessment visits other than the cardiologist evaluation at inclusion, and at 6 months, 17% had one visit, and 5% had more than one visit. During this period, 45% of participants had no telephone consultations beyond the protocol-specified call at 8 weeks, 35% had one consultation, and 20% needed more than one consultation. Ninety percent of participants had no unscheduled visits during standardized telemonitored medical titration until OMT was achieved. Eight percent of participants had one visit, and 2% had more than one visit. During medical titration, around two-thirds of participants (63%) had no phone consultations besides study protocol, 28% had one phone consultation, and 9% had more than one phone consultation. Besides titration-specific contacts, participants had routine follow-ups for SWEDE-HF registration with HF nurses at inclusion and after achieving OMT (typically 2–6 months after inclusion). No medical decisions were made at these visits. Some participants had cardiologist visits post-OMT for evaluation. HBM was generally appreciated, promoting security, quick nurse access, and better diagnosis understanding.

### Safety

No serious adverse events occurred during standardized titration using HBM. The HBM system’s alarm thresholds were SBP <90 or >160 mmHg, diastolic blood pressure <50 or >100 mmHg, pulse <50 or >100 b.p.m., and a weight gain of >2 kg within 48 h. The most common adverse event was bradycardia, occurring in eight patients (13.3%) and was associated with BB therapy. Renal impairment was the second most frequent adverse event, reported in four participants (7%), followed by hypotension in three patients (5%). ACE inhibitor-related dry cough was observed in three participants (5%), all of which resolved following a switch to ARB (*Table 3*). During follow-up, severe renal impairment without full recovery occurred in two participants: one case was linked to contrast use during coronary angiography, and the other was associated with cytotoxic cancer treatment and several contrast-using modalities. Less common adverse events are summarized in *Table 3*.

**Table 3:** Adverse events during standardized GDMT titration and 6-month follow-up

Adverse Event	Count N	Percentage (%)
Bradycardia^a^	8	13
Renal impairment^b^	4	7.0
Hypotension^c^	3	5.0
Cough	3	5.0
Vertigo	2	3.3
Gynecomastia	1	3.3
Hair loss	1	1.7
Hyperkalemia^d^	1	1.7
Elevated liver enzymes	1	1.7
Impotence	1	1.7
Urinary tract infections	1	1.7

^a^Heart rate <50 b.p.m.

^b^P-Creatinine increase >50% with eGFR < 60 mL/min/1.73 m^2^.

^c^SBP <90 mmHg.

^d^P-Potassium >5.5, needing intervention.

## Discussion

The TELEFASTER-HF study presents a promising approach for rapidly achieving target GDMT doses or OMT in patients with HFrEF. To our knowledge, this is the first study using HBM in combination with a standardized medication titration protocol incorporating all four GDMTs. The concept is based on a reversed titration strategy compared with the traditional approach. During traditional titration of GDMT, repeated individual clinical decisions carry the risk of slowing the titration process, potentially contributing to treatment inertia. Instead, we propose a standardized pre-specified titration approach within 8 weeks. Reasons to depart from the pre-specified scheme were objective and based on vital parameters, safety blood sampling, and/or side effects potentially impeding further titration at the pre-specified pace. This study shows that this is achievable with the use of HBM, providing new insights to the field. The development of digital solutions to optimize GDMT implementation is progressing rapidly. These include digital consultations, HBM, clinical decision support systems, and implantable devices. How these can be utilized and combined to achieve the best results for heart failure patients remains unclear, and various strategies are currently being tested worldwide to address these questions.^19^ Recent studies have confirmed positive results using HBM for GDMT titration. Artanian *et al*. and Brahmbhatt *et al*. reported that HBM with structured titration was safe and more effective in reaching OMT than traditional titration approaches.^16,17^ However, the time to OMT was considerably longer (3,42 months) than here described, and every titration step included a checkpoint for decision-making by a cardiologist. Furthermore, very few patients reached target GDMT doses.^17^ The ADMINISTER study made an important contribution to how digital tools can enhance GDMT utilization, resulting in improved GDMT scores and OMT rates. Unlike our study, no standardized protocols for titration were used, but the approach using guideline-based digital summaries to support clinical decision-making, facilitates a more consistent titration process with fewer personal opinions influencing medical decisions.^18^ With the help of a standardized titration, most participants in our study reached OMT within 8 weeks after inclusion, and all participants reached OMT within 6 months. Furthermore, more than 40% of participants in the study reached target doses of all GDMT medications, and a definite majority reached target doses of both BB and RASi. To our knowledge, this high level of achieved GDMT and the pace of titration have only been matched in one previous study. However, that study required numerous clinical visits and controls, making it challenging to implement in routine clinical practice.^12^ The TELEFASTER-HF concept is easy to implement and has the potential to streamline the titration process, freeing up time for healthcare providers while promoting more equitable care for HF patients. Throughout titration and the study period, there was little need for in-clinic assessment visits or phone consultations. Most participants’ questions and healthcare personnel instructions regarding individualized titration adjustments were effectively handled through the chat function. Importantly, we found no major safety issues associated with using a standardized titration approach, and the adverse events were few. The HBM system, combined with regular blood work, effectively detected any adverse events, allowing the HF clinic to take timely and appropriate action.

For all four GDMT medications, we could see significant dosing increases up to week eight. Between week eight and the 6-month follow-up, a modest but significant decrease in dosing was observed for BB, RASi, and MRA. In some cases, this may be due to participants not achieving OMT during the initial standardized rapid titration phase, necessitating adjustments to their GDMT doses after the first 8 weeks. While initial side effects may be tolerable during initial rapid titration, persistent side effects can become intolerable over time. In most cases, the doses were adjusted after achieving OMT. This is an important observation and highlights the need for close follow-up of these patients during the first period after the HF diagnosis. HBM facilitates close contact with the patients and may enable maintaining high doses of GDMT during the first period after diagnosis. HBM may also increase compliance, which is demonstrated in this study by the overall high GDMT persistence at 6 months despite the decrease after 8 weeks. Importantly, a reduction in HF admissions has also been shown in recent studies on HBM in HF patients, which further highlights the potential of this approach.^13,15^ In this study, the participants did not continue with HBM after the 6-month follow-up visit, and a prolonged HBM strategy might have further effects regarding health care burden and medication adherence.

### Limitations

This was a feasibility study with a proof-of-concept design, and the results must be interpreted cautiously. The study design has inherent limitations, including the risk of selection bias. The number of patients and the fact that only patients with up to stage IIIa kidney failure were included further limit the generalization of the results. Some unintentional selection bias related to age and frailty is possible, as the eGFR threshold and early screening may have excluded older HF patients. Additionally, the requirement for digital access and proficiency may have further narrowed inclusion. Although enrolment was consecutive, these criteria suggest the cohort represents a more capable segment of the HFrEF population. These limitations warrant caution when interpreting the generalizability of the results. While this introduces potential bias, it also offers notable advantages. It efficiently identifies a subgroup of patients capable of safely undergoing a standardized GDMT titration using HBM. Furthermore, a strength of the study was the consecutive inclusion of patients with few exclusion criteria, seamlessly integrating the study design into clinical care without significant obstacles.

## Conclusion

This study introduces a novel approach to the management of GDMT titration. Supported by telemonitoring, our data show that a standardized titration process is promising in terms of both efficacy and safety. Numerous previous reports have highlighted the challenges of implementing HF medication guidelines, particularly when each dose titration step requires individual decisions from healthcare practitioners and clinical visits. This study offers potential solutions to these challenges by demonstrating the feasibility of standardizing GDMT up-titration by protocol and incorporating HBM. The results of this study now need to be confirmed in a larger randomized study.

### Clinical perspectives and future studies

While the cohort examined represents a selected population, the concept might be adapted to include a broader range of patients, possibly with the use of more individualized standardized algorithms for titration. This would require a larger randomized study in which subgroup analyses could be performed, for example, for older patients, frail patients, and patients with more pronounced renal failure. A longer follow-up period with a larger group of patients could also help us to better identify and analyse the factors contributing to a gradual reduction in medication doses over time. Potential future improvements could involve combining standardized titration supported by HBM with digital consults and clinical decision support, particularly when patients cannot follow the pre-specified titration pace or when adverse effects occur during follow-up. This approach might enable more patients to achieve OMT faster and maintain higher GDMT doses over time. Another important consideration is the impact on health economics and resource utilization. In this study, we observed that participants had few physical visits and telephone consultations during titration. However, this study cannot answer the question of how this compares to the standard of care, but our results suggest an advantage in the standardized design of the titration process, which may reduce the need for healthcare contacts. This proof-of-concept study lays the groundwork for future larger randomized trials that will provide more robust evidence to validate our conclusions, explore additional in-depth questions, and further advance the concept.

## Supplementary Material

ztaf062_Supplementary_Data

## Data Availability

The data underlying this article will be shared upon reasonable request to the corresponding author.
