# Supplementary material for: Telemonitored standardized titration for heart failure with reduced ejection fraction, an open clinical cohort study
Source: Eur Heart J Digit Health. 2025 Jun 5;6(5):897–906. doi: 10.1093/ehjdh/ztaf062 (PMC12450508; doi:10.1093/ehjdh/ztaf062)
Supplement: ztaf062_Supplementary_Data [file ztaf062_supplementary_data.zip › supplementary table 1.docx]

Supplementary table 1. GDMT dosing definitions

| **Drug** | Beta-blocker  (BB) | | Sodium-glucose cotransporter 2 (SGLT-2) inhibitors |
| --- | --- | --- | --- |
| **Daily**  **Dosage** | None | 0mg | 0mg |
|  | ≤ 50% of target dose | 0mg >Metoprolol ≤ 100mg  0mg > Bisoprolol ≤ 5mg  0mg > Carvedilol ≤ 25mg (if <85kg)  0mg > Carvedilol ≤ 50mg (if >85kg) | - |
|  | >50% - < target dose | 100mg > Metoprolol < 200mg  5mg > Bisoprolol < 10mg  25 > Carvedilol < 50mg (if <85kg)  50mg > Carvedilol < 100mg (if >85kg) | - |
|  | Target dose or more | Metoprolol ≥ 200mg  Bisoprolol ≥ 10mg  Carvedilol ≥ 50mg (<85kg)  Carvedilol ≥ 100mg (>85kg) | Dapaglifozin ≥ 10mg  Empaglifozin ≥ 10mg |

| **Drug** | RASi  (renin-aldosterone system inhibitors) | | MRA (mineralocorticoid receptor antagonist) |
| --- | --- | --- | --- |
| **Daily Dosage** | None | 0mg | 0mg |
|  | ≤ 50% of target dose | 0mg >Ramipril ≤ 5mg  0mg > Enalapril ≤ 10mg  0mg > Losartan ≤ 75mg  0mg > Candesartan ≤ 16mg  0mg > Valsartan ≤ 160mg  0mg > Entresto ≤ 98/102mg | 0mg > Spironolactone ≤ 25mg  0mg > Eplerenone ≤ 25mg |
|  | >50% - < target dose | 5mg > Ramipril < 10mg  10mg > Enalapril < 20mg  75mg > Losartan < 150mg  16mg > Candesartan < 32mg  160mg > Valsartan < 320mg  98/102mg > Entresto < 194/206mg | - |
|  | Target dose or more | Ramipril ≥ 10mg  Enalapril ≥ 20mg  Losartan ≥ 150mg  Candesartan ≥ 32mg  Valsartan ≥ 320mg  Entresto ≥ 194/206mg | Spironolactone ≥ 50mg  Eplerenone ≥ 50mg |
