# Supplementary material for: Telemonitored standardized titration for heart failure with reduced ejection fraction, an open clinical cohort study
Source: Eur Heart J Digit Health. 2025 Jun 5;6(5):897–906. doi: 10.1093/ehjdh/ztaf062 (PMC12450508; doi:10.1093/ehjdh/ztaf062)
Supplement: ztaf062_Supplementary_Data [file ztaf062_supplementary_data.zip › supplementary table 2.docx]

Supplementary table 2. TELEFASTER-HF weekly digital health questionary

| Questions | 0 point | 1 point | 2 point | 3 point |
| --- | --- | --- | --- | --- |
| How do you experience your swelling in your lower legs and ankles today? | No swelling at all | Mild swelling | Moderate swelling | Pronounced swelling |
| How do you experience your fatigue today? | Completely unaffected | Fatigue with more than moderate exertion | Fatigue through light exertion | Fatigue at rest |
| How do you experience your shortness of breath today? | Completely unaffected | Shortness of breath with more than moderate exertion | Shortness of breath with light exertion | Shortness of breath at rest |

Weekly questionary with a scoring system where scoring more than 7 points triggers a warning in the HBM system. Participants were asked complete the questionary at least once per week, but the participant could choose to complete it more frequently.
