# Supplementary material for: Telemonitored standardized titration for heart failure with reduced ejection fraction, an open clinical cohort study
Source: Eur Heart J Digit Health. 2025 Jun 5;6(5):897–906. doi: 10.1093/ehjdh/ztaf062 (PMC12450508; doi:10.1093/ehjdh/ztaf062)
Supplement: ztaf062_Supplementary_Data [file ztaf062_supplementary_data.zip › supplementary table 4.docx]

# Schedule 2. TELEFASTER-HF STUDY: The table below shows the titration schedule for patients on either Metoprolol 50 mg DD, Bisoprolol 2,5 mg DD, or Carvedilol 6,25 mg DD

| Day 1 | Metoprolol/Bisoprolol/Carvedilol | 50 mg 1x2/2.5 mg 1x2/6,25 mg b.i.d |
| --- | --- | --- |
|  | Dapagliflozin/Empagliflozin | 10 mg b.i.d |
|  |  |  |
| Day 8 | Enalapril/Ramipril/Sacubitril-Valsartan/Candesartan | 2,5 mg b.i.d/1,25 mg b.i.d/24/26mg b.i.d/4 mg o.d |
|  |  |  |
| Day 15 | Metoprolol/Bisoprolol/Carvedilol | 200 mg b.i.d/10 mg o.d/12,5 mg b.d |
|  |  |  |
| Day 18 | Spironolakton/Eplerenon | 25 mg o.d* |
|  |  |  |
| Day 22 | Enalapril/Ramipril/Sacubitril-Valsartan/Candesartan | 5 mg b.i.d/2,5 mg b.i.d/49/51mg b.i.d/8 mg o.d |
|  |  |  |
| Day 29 | Carvedilol | 25 mg b.i.d |
|  |  |  |
| Day 36 | Enalapril/Ramipril/Sacubitril-Valsartan/Candesartan | 10 mg b.i.d/5 mg b.i.d/97/103 mg b.i.d/16mg o.d |
|  |  |  |
| Day 43 | Spironolakton/Eplerenon | 50 mg o.d |
|  | Carvedilol | 50 mg b.i.d** |
|  |  |  |
| Day 50 | Candesartan | 32 mg o.d |
|  |  |  |
| *Spironolakton/Eplerenon should be increased to 50 mg o.d on day 18 in patients already on 25 mg o.d.  **Considered in patients weighing >85 kg | | |
